# Dr. Hall, on Catarrh

**Published:** 1805-03-01

**Authors:** R. Hall

**Affiliations:** Clement's Inn


GENTLEMEN,

NOTWITHSTANDING the ample discussion which has taken place relative to the nature and character of the late influenza, there still, I observe, exists a considerable difference of opinion among practitioners, not only with regard to the treatment of this disease, but also respecting the mode of its propagation. If, therefore, you deem the arguments and evidence brought forward in the following cursory observations, which were written during the decline of that epidemic, without any view at the time to publication, as tending in the least degree to elucidate the subject, I have to request that you would give them a place in your useful Journal.

I am, &c.

*Clement's Inn, Jan.* 4, 1805. R. HALL.

---

The nature and origin of febrile contagion, have, in every age, deservedly attracted the attention of physicians, and various doctrines have been maintained by different authors on this subject. Hippocrates himself, although he certainly marks with care the changes of seasons, in his account of diseases, nevertheless seems to favour the opinion that all epidemics are the offspring of a pestilential constitution of the air, since the τὸ θεῖον, to which he ascribes so much influence in their production, can be only understood to refer to some occult principle in the atmosphere.

Fracastorius, a writer of the fifteenth century, appears also to have entertained sentiments nearly similar on this subject; for, in speaking of the sudor Anglicanus, which, from the accounts transmitted to us, appears to have had a striking resemblance to epidemic catarrhs of modern times, he observes, "In Britannia insula genus est pestilentis febris, et contagiosae . . . . est autem non contagiosa solum de uno in alium, sed vagatur etiam de civitate in civitatem. Quapropter in vitio aëris praecipue enasci censendum est."

Allied to these opinions is the doctrine of the celebrated Sydenham, that certain constitutions of the atmosphere, produced from occult causes certain diseases, and favoured the diffusion of others. This doctrine he applied not only to the plague, and other exanthematic affections, such as the small-pox, measles, &c. but also to acute contagious diseases in general when prevailing in an epidemic form. Such a doctrine seems, however, repugnant to the universal experience of medical practitioners, and is evidently the result of a partial and narrow view of the subject. But, independently of direct facts, the inferences deducible from this opinion are, in our apprehension, equivalent to a *reductio ad absurdum*, for, if the doctrine were well founded, it would inevitably follow, that no human power could prevent the propagation of infection, and all attempts by ventilation, separation, and other means to arrest its progress, would prove altogether abortive. Fortunately, however, experience has not only proved the utility of such measures in controlling the destructive agency of contagious virus, but that every kind of contagion with which we are acquainted, is so circumscribed in its sphere of action, as to be incapable of producing any noxious effects, except at a small distance from its source.

Other writers, renouncing the agency of any occult principle, endeavour, on the contrary, to explain the rise and progress of all epidemics from the sensible qualities of the air; but although it must be admitted, that sudden alterations of temperature, or rather the stimulus of heat, acting on excitability much accumulated from previous exposure to cold, produce great effects on the living system, yet, when we consider the gradual progress, the circumscribed range, and still more especially the prevalence of such diseases in all kinds of seasons, and in very different states of the weather, it must we conceive be acknowledged, that, whatever share such changes may remotely have in the production of pestilential maladies, they are propagated and diffused by the influence of other means than mere alternations of temperature, or any peculiar state of the atmosphere.

The origin of contagion is unquestionably a subject involved in considerable obscurity, and until farther advances be made in chemical physiology, may perhaps still continue to elude investigation. From a variety of facts, however, it appears sufficiently probable, that all pestilential maladies arise from a specific matter generated in the animal system, in consequence of diseased action, and that they are afterwards communicated from one person to another, in proportion to the degree of concentration in the poison, and the susceptibility of the individuals exposed to its influence.

The

The application of the same noxious powers, originally productive of any given contagion, may doubtless at all times individually produce it, under similar circumstances; although there seems reason to believe that this is the case in comparatively few instances; yet, without admitting this position as a fact, how shall we explain the occurrence of typhus, which is indubitably contagious, in persons at a period when, on the strictest investigation, the disease could be traced to no infectious source whatever?

What has been here advanced, respecting pestilential diseases in general, appears to us strictly applicable to the present influenza; for although various facts and arguments have been adduced to disprove the infectious quality of this malady, yet, so far as attentive observation has enabled us to form any judgment on the subject, we are decidedly of opinion, that it is in its nature truly contagious, and alike communicable, as the poison of typhus, scarlatina, &c. from one individual to another.

The reasons which principally induce us to conclude, in common with many other practitioners, that this disease is propagated by individual contagion, are:

1st. From having, in several of the cases which fell under our own observation, been able distinctly to trace previous communication between the patient, and some infected person or place wherein the disease existed.

2dly. From the safety of several families, that remained wholly in a state of seclusion during the prevalence of the epidemic.

3dly. From its attacking persons in succession, living in the same house or family.

4thly. From its spreading, like other infectious diseases, more especially among those exposed, by their more free intercourse with each other, to the influence of the contagion.

Without, however, taking into consideration these and various other facts, which might be adduced as affording the most satisfactory evidence of the propagation of this disease by human contagion, how, on the supposition of its proceeding from a morbid state of the atmosphere, did it not affect different countries at the same period? Or how, if, according to the advocates of this opinion, the mass of air were so vitiated, can we explain the progressive course of the disease? Surely, a cause so universal should have produced effects more nearly simultaneous. Whereas, on the contrary, a marked interval occurred not only between its appearance in one county and

O 2 another, another, but even in different towns, and in different individuals of the same family. Why, moreover, were individuals exempted, who, though not confining themselves within doors, yet sedulously kept apart from persons or places, wherein the disease existed? How, on the same principle, can we explain the appearance of this epidemic, first in sea-port towns and in large cities, and its gradual diffusion afterwards in every direction through the circumjacent country? Or how, in the present case, can the air act as an agent in disseminating the infectious virus, when it has been fully demonstrated, that all other febrilizing poisons are rendered innoxious, either by diffusion, or chemical solution in the atmosphere.

These and many other similar questions cannot, it is obvious, be satisfactorily answered by those who ascribe the disease in question to a morbid constitution of the atmosphere; whilst, on the contrary, all of them may be easily accounted for by admitting the disease in its origin and progress to depend on human contagion.

Nothing can be more fallacious in medical science, than to build any theory, or to establish any principle, on a few detached facts, inaccurately stated, and ill understood; yet, in the question at issue, it has always appeared to us that the advocates for the non-contagious nature of the influenza have been betrayed into this error, by founding their conclusions on the exemption of individuals, who, though exposed to the action of the contagion, remained free from the complaint: but, it surely does not hence follow, that the epidemic in question is not of a contagious nature, since similar cases of exemption not unfrequently occur on exposure to the variolous, and other infectious poisons, which are yet universally allowed to be contagious. If, therefore, a mass of positive testimony can be adduced to prove the contagious character of epidemic catarrh, little regard is due to any negative facts or exceptions, since, were such a mode of argumentation admissible, it might be proved that no disease whatever is contagious.

Another source of fallacy, respecting the character of this disease, seems to have arisen from a want of due attention to the distinction between catarrh and influenza, which, it must be confessed, unless in strongly marked cases, and without a strict inquiry into the symptoms, and the previous situation of the patient, it is not always easy to ascertain. Besides as, from the testimony of practitioners themselves, it appears that the idea of the influenza, during during its prevalence, took such complete possession of the mind as to be frequently associated not only with catarrhal complaints, but with almost every other indisposition to which it had the most distant resemblance, it seems natural to infer, that these circumstances would frequently lead to an erroneous judgment respecting the nature of the malady under consideration.

Several instances are recorded of the same patient having been re-attacked by the influenza, during the same season; and as it has been supposed, that specific contagions can affect individuals only once during life, this circumstance has been urged as a farther confirmation of the non-contagious nature of this malady. Now, although the above doctrine may hold true in general, with regard to measles, small-pox, &c. it will not apply to other contagious diseases, as typhus, scarlatina, &c. to which, after some time, the same susceptibility returns as at first; admitting even, therefore, that in a few individual cases, a second attack of the influenza may really have occurred, yet this is no more than has, in some instances, been observed in other diseases allowed to depend on specific contagion, and can afford no convincing argument in favour of the non-contagious nature of this disease. We ourselves never witnessed a second accession of influenza; that is, any case where, after a complete recovery, the disease recurred with the same characteristic symptoms as at first; but we have frequently met with instances, in which the symptoms became so aggravated by neglect and mismanagement, as to assume the appearance of a new attack. We are, however, far from asserting, that in no instance whatever did any person experience a recurrence of this disease; but we may venture to affirm that it is a circumstance of more rare occurrence than some practitioners seem willing to admit, and that relapses from inattention or neglect, during the period of convalescence, must have frequently given occasion to this mistake.

Of all epidemics with which we are acquainted, the influenza is the most remarkable for its wide and extensive range, seldom appearing in any one country of Europe, without being successively transferred to every other part of it, as well as frequently to other quarters of the globe. The supposition of this malady being a contagious disease, communicable by personal intercourse, the opponents to infection likewise allege, does not accord with its wide and rapid extension, since other contagious maladies, they observe, are not propagated with the same degree of rapidity that marked its progress. This argument, however, cannot in our opinion furnish any valid objection, tending to disprove the contagious character of the disease; for when patients labour under any epidemic affection avowedly contagious, they are for the most part, through necessity, subjected to confinement; and the propagation of infection is thereby restrained: whereas, in the influenza, the majority of those who were attacked by it, experienced so slight an indisposition, that continuing to intermix with society at large, they disseminated the contagion more widely and generally than in other epidemics.

In contagious diseases, it is besides well known, that the poison, after its reception into the system, remains for a longer or shorter period in a dormant state, before exciting any perceptible commotion in the animal economy. From this circumstance, as well as from the invisible nature of the miasmata, it is indeed extremely difficult in many cases, satisfactorily to trace the communication of the contagion; yet, in our opinion, the facts recorded respecting the rise, progress, and declension of this and similar epidemics, with their prevalence under different degrees of temperature, and in very different states of the weather, furnish as striking evidence as the nature of the case will admit, that the influenza is, in its origin and progress, wholly dependent on human contagion.

We cannot affirm, from our own experience, whether the influenza could be imparted by clothes or fomites; but reason and analogy, as well as several facts recorded in the history of this affection, tend to support the affirmative of this proposition, and induce us to believe, that the infection may occasionally be conveyed by such intermedia, in a sufficient degree of concentration to give rise to the disease.

As domestic animals, it is well known, frequently suffer as well as man, under the operation of analogous agents, we endeavoured, with a view to obtain farther light as to the nature of the disease, and its remote causes, likewise to ascertain, whether any of them had been subjected to a similar malady; but we have not been able to procure any information on this subject deserving of attention. Mr. Du Gard, in the Medical and Physical Journal for Sept. 1804, has recorded the case of a boy who appears to have caught the influenza from caressing a cat ill of this disorder. What seems to us the converse of this case immediately fell under our own observation. A cat carefully kept secluded, for a particular purpose, from every other other animal, within a few hours after being fondled by a person labouring under the disease, was attacked by a complaint, which evidently seemed to be an inflammatory affection of the Schneiderian membrane. As the animal had always been confined in a room of a very uniform temperature, and never exposed to any vicissitudes of heat and cold, it is not improbable that the catarrhal affection was in this case communicated from the human to the brute animal. But, however analogical reasoning may tend to fortify such an inference, yet we are fully aware that this opinion requires farther confirmation, and that without an enlarged induction of facts, we can conclude nothing decisive on this subject.

It would be here wholly unnecessary to enter into any history of the symptoms of this affection, since these have been sufficiently described by the numerous writers on this important subject. With respect to the essential character of the disease, the sentiments of practitioners appear to be no less dissimilar than with regard to its contagious nature: this, however, is a question of the utmost importance to ascertain, as much mischief may arise by not distinguishing its occasional varieties, produced by difference of age, sex, temperament, &c. from the permanent and specific character of the disease. For our own part, after an attentive observation of its prevailing symptoms, in the very few cases which fell under our own observation, we are inclined to agree with those practitioners, who consider it to be of an inflammatory nature; for although the symptoms in this, as in former epidemics of the same kind, varied, not only in different individuals, but likewise in their degree of violence and duration, as well as with respect to the order in which they occurred, yet the disease never lost its appropriate character, and indeed, in the majority of cases, appeared only to differ in degree from common catarrh. We were farther confirmed in this opinion, by observing the greater or less local irritation that always prevailed in the pulmonary organs, as well as by the disease readily yielding to a cooling plan of treatment. As to the extreme debility, which is considered by some practitioners as constituting the leading and pathognomic symptom in this affection, we are inclined to think, that it was at least greatly aggravated, if not often induced by the injudicious employment of stimulants in the incipient stage of the disease, frequently even before recourse was had to medical assistance. In our limited practice, this was no very unusual occurrence; in one case in particular,* that of Mr. Gibson, a very ingenious artist residing at Hampstead, the mischief resulting from such conduct, and the beneficial effects of a contrary mode of treatment were so evident, as to strike even the patient himself.†

In the slighter forms of the disease, nothing more was necessary than an attention to regimen, and the proper regulation of the bowels; in more violent cases, however, especially if attended with nausea or sickness, emetics were found useful. When local pains, accompanied with impeded respiration, prevailed, they readily yielded to the application of blisters, or repeated friction with rubefacients. Opiates generally proved hurtful in the first stage of the complaint, during the continuance of local and general excitement; but, towards its decline, when the febrile symptoms had subsided, they were found extremely beneficial, not only in mitigating the severity of the cough, but in promoting expectoration, and supporting a determination

---

* In this case, which was distinctly traceable to personal communication, the infection came into action within forty-eight hours after it had been received.

† During the prevalence of epidemic catarrh in 1782, I had an opportunity of witnessing the beneficial effects of a cooling regimen in this disease on a pretty extensive scale in the south of Scotland. Among that class of inhabitants, who are not in the habit of calling in medical aid, except on very urgent occasions, it was a favourite custom, on being attacked by influenza, to drink at going to bed a large draught of the coldest spring water they could procure, strewed over with a handful of oatmeal. This remedy, as may well be supposed, generally operated to produce a diaphoresis, or sweat, and aided by atmospheric exposure, and a meagre diet, to which such patients are necessarily subjected, seldom failed speedily to put an end to the complaint. Whereas, on the contrary, amongst the more opulent classes, who indulged themselves in warm apartments, and in the use of a heating regimen, the disease was, for the most part, protracted to a considerable length. Whilst, to the unenlightened mind this remedy appeared to possess some occult virtue, to the medical practitioner it must be obvious, that it acted merely, like other cooling fluids, by diminishing preternatural heat, and repressing systematic irritation.

A fact to the same purpose, and which I recollect struck me very forcibly on reading it some years ago, is related by Dr. Rutty in his History of the Weather and Seasons during the Continuance of the Catarrhal Fever among the Horses in 1760, and which, in its origin and progress, appears from his account, to have been subject to the same laws as the influenza among the human species. A great many of these animals fell a sacrifice to too warm a regimen, particularly in the barracks at Dublin, where they were kept closely crouded in the stables; here they lost thirty-eight out of two hundred and fifty: whilst at a stand of chaise horses, subjected to more exercise, and a cooler regimen, the proportion of deaths was only two out of thirty-six.

mination to the skin. When however the cough proved more obstinate than usual, and, at the same time, was accompanied with general irritability, quick and hard pulse, dyspnœa, &c. no medicine was found more effectual in combating these symptoms than small doses of tincture of digitalis, either exhibited by itself, or in conjunction with opiates. By such means, and the occasional employment of other auxiliaries, the complaint never failed to be speedily overcome.

---

